# Evolutionary Dynamics and Functional Bifurcation of the C2H2 Gene Family in Basidiomycota

**DOI:** 10.3390/jof11070487

**Published:** 2025-06-27

**Authors:** Chao Duan, Jie Yang

**Affiliations:** 1 Institute of Cotton Research, Shanxi Agricultural University, Yuncheng 044000, China; 2 Shanxi Institute for Functional Food, Shanxi Agricultural University, Taiyuan 030031, China; mu.yang633@163.com

**Keywords:** C2H2 genes, Basidiomycota, phylogenomic classification, gene family evolution, synteny network

## Abstract

This study performed a phylogenomic analysis of the C2H2 gene family across 30 Basidiomycota species, identifying 1032 genes distributed across six evolutionary clades (Groups I–VI). Functional diversification and lineage-specific expansions were observed: Group II (37.1%) formed a conserved core, while wood decayers (e.g., *Schizophyllum commune*) and edible fungi (e.g., *Pleurotus ostreatus*) exhibited clade-specific expansions in Groups III and V, respectively. Physicochemical profiling revealed an acidic bias in Agaricomycotina proteins (pI 4.3–5.8) compared to alkaline trends in pathogens (Ustilaginomycotina/Pucciniomycotina; pI 8.3–8.6). Comparative genomics indicated that saprotrophs retained long genes (12.4 kb) with abundant introns (mean = 6.2/gene), whereas pathogens exhibited genomic streamlining (introns ≤ 2). Synteny network analysis revealed high ancestral conservation in core clusters (Cluster_1–2: 58% homologs) under strong purifying selection (Ka/Ks = 0.18–0.22), while peripheral clusters (Cluster_Mini) approached neutral evolution (Ka/Ks = 0.73). This study reveals stage-specific expression dynamics of 17 C2H2 zinc finger genes in *Sarcomyxa edulis*, highlighting their roles in coordinating developmental transitions (e.g., *SeC2H2_1* in low-temperature adaptation, *SeC2H2_7/12* in primordia initiation, and *SeC2H2_8/9/13* in fruiting body maturation) through temporally partitioned regulatory programs, providing insights into fungal morphogenesis and stress-responsive adaptation. These findings underscore the dual role of C2H2 genes in sustaining conserved regulatory networks and facilitating ecological adaptation, providing new insights into fungal genome evolution.

## 1. Introduction

C2H2 zinc finger proteins, one of the largest transcription factor families in eukaryotes, are essential for cellular differentiation, stress response, and secondary metabolism [1]. In fungi, these proteins regulate essential processes such as host–pathogen interactions (e.g., effector proteins in Magnaporthe oryzae), lignocellulose degradation (e.g., laccase expression in white-rot fungi), and morphological development (e.g., hyphal formation in yeast) [2,3]. However, systematic studies on Basidiomycota, a group with greater species diversity and more complex ecological functions, remain scarce. Specifically, the evolutionary patterns, functional diversification, and ecological relevance of C2H2 genes within this lineage remain underexplored.

Recent genomic studies have revealed distinct evolutionary traits in Basidiomycota: Agaricomycotina saprotrophs expand gene families for complex substrate metabolism [4,5], while Ustilaginomycotina/Pucciniomycotina pathogens streamline their genomes to optimize host infection [6,7]. These observations suggest that the C2H2 gene family may function as a pivotal element in basidiomycete functional diversification. However, several critical gaps persist: (1) comparative genomics across subphyla are lacking; (2) the relationship between duplication mechanisms and selection pressures remains unclear; (3) the interplay between conserved regulatory networks and adaptive evolution has not been quantified.

This study presents the first phylogenomic analysis of the C2H2 gene family across 30 representative Basidiomycota species, combining phylogenetic reconstruction, synteny network analysis, selection pressure evaluation, functional element prediction, and transcriptional expression analysis. By addressing three key questions—(1) How is the C2H2 family evolutionarily structured within Basidiomycota? (2) How do subphylum-specific gene architectures and regulatory elements correlate with ecological adaptation? (3) What are the expression differences among members of different gene families at different growth stages?—this study aims to clarify the evolutionary principles governing fungal transcriptional networks and provide insights into how genomic plasticity drives ecological niche differentiation.

## 2. Materials and Methods

### 2.1. Data Retrieval and Quality Assessment

Genomic datasets were curated using a two-tier filtration protocol that prioritized assembly completeness and taxonomic representation within Basidiomycota. Initial candidate genomes were retrieved from NCBI GenBank (https://www.ncbi.nlm.nih.gov/, accessed on 13 February 2025) in FASTA format. Assembly completeness was assessed with BUSCO v5.4.3 using the fungi_odb10 database [8], retaining genomes with ≥82% complete BUSCO scores. Taxonomic balance was achieved by selecting 30 representative genomes from all three Basidiomycota subphyla: 24 Agaricomycotina (wood decayers/saprobes), 3 Pucciniomycotina (rust fungi), and 3 Ustilaginomycotina (smut fungi). Detailed metadata, including NCBI accession numbers, taxonomic classification, genome size, and BUSCO metrics, are provided in Supplementary Table S1.

### 2.2. Identification of C2H2 Gene Family Members

The Hidden Markov Model (HMM) profile for the C2H2 zinc finger domain (PF00096) was obtained from the Pfam database [9,10]. Candidate C2H2 proteins were identified using the hmmsearch command in HMMER (v3.3.2) with parameters—incdomE 0.01—domtblout out.txt input.fa to scan protein sequences of each species. To ensure accuracy, all candidate sequences were further validated for C2H2 domains using InterProScan (v5.56-89.0) [11] and the Conserved Domain Database (CDD) [12]. Sequences lacking the canonical C2H2 motif (X2-C-X2,4-C-X12-H-X3,4,5-H) were discarded [13]. Physicochemical properties (e.g., molecular weight and isoelectric point) were calculated using Bio::Tools::pICalculator (https://metacpan.org/pod/Bio::Tools::pICalculator, accessed on 15 February 2025) and ProtParam. Subcellular localization predictions were made with Euk-mPLoc 2.0 (http://www.csbio.sjtu.edu.cn/bioinf/euk-multi-2/, accessed on 16 February 2025).

### 2.3. Gene Structure Analysis of C2H2 Family

Exon–intron structures of C2H2 genes were determined by mapping genomic coordinates from GFF3 files to corresponding coding sequences [14].

### 2.4. Phylogenetic and Evolutionary Analysis of C2H2 Genes

A species tree for the 30 basidiomycete genomes was constructed using OrthoFinder (v2.5.4) [15]. Protein sequences were aligned with MUSCLE (v5.1), and phylogenetic trees were generated using FastTreeMP (v2.1.11) under default parameters. Tree visualization and annotation were performed with the Interactive Tree of Life (iTOL, https://itol.embl.de/, accessed on 23 February 2025) [16]. Synteny analysis was conducted with JCVI (v1.1.13) [17], and duplicated genes were classified using the duplicate_gene_classifier pipeline. Syntenic blocks specific to the C2H2 family were filtered and visualized using Cytoscape (v3.9.1). Non-synonymous (Ka) and synonymous (Ks) substitution rates were calculated with KaKs_Calculator (v2.0) using the YN method [18].

### 2.5. Promoter Cis-Regulatory Element Analysis

DNA sequences spanning 2000 base pairs (bp) upstream of the C2H2 gene translation start site (ATG) were extracted from reference genomes. Putative cis-regulatory elements were identified using the PlantCare database (http://bioinformatics.psb.ugent.be/webtools/plantcare/html/, accessed on 25 February 2025) [19].

### 2.6. Expression of C2H2 Zinc Finger Genes in Transcriptomes of Sarcomyxa edulis

The *Sarcomyxa edulis* strain (No. 2016120327) was cultivated under standard conditions. Samples were collected at six critical developmental stages: B1: mycelium occupying half the cultivation bag; B2: mycelium fully colonizing the bag under low-temperature stimulation; B3: primordia initiation stage; B4: early primordia formation; B5: mycelium at harvest maturity; B6: mature fruiting body. For comprehensive experimental methods, refer to our earlier publication [20].

## 3. Results

### 3.1. Systematic Identification and Classification of C2H2 Genes in Basidiomycota

**Figure 1:**
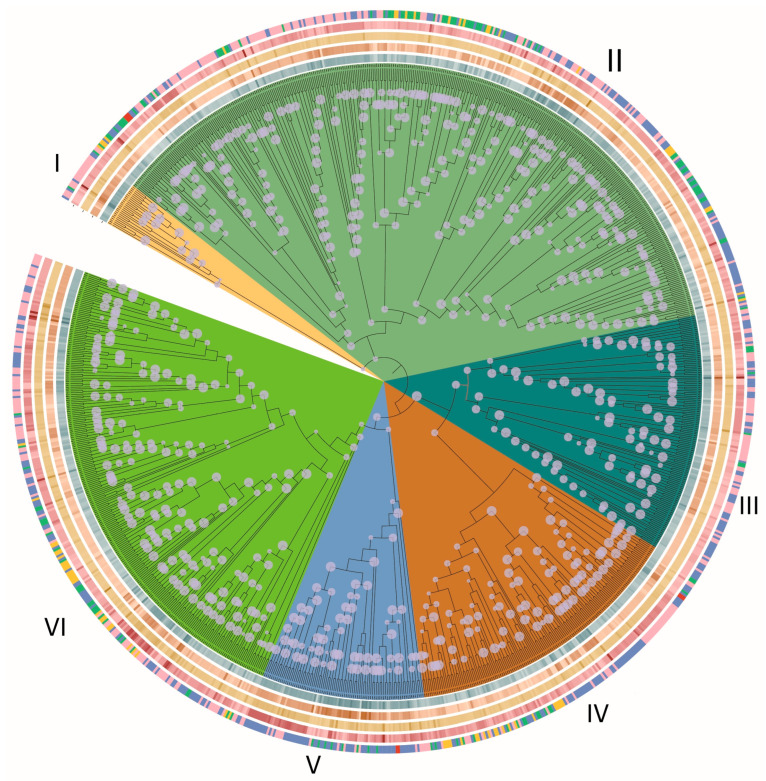
Phylogenetic analysis of fungal C2H2 genes with structural and evolutionary features. Note: From outer to inner rings, the following is observed: gene length, protein length, intron length, intron number, and duplication types. Color intensity correlates with value magnitude. Duplication types are as follows: dispersed (blue), singleton (pink), tandem (yellow), proximal (green), and segmental (red). Nodes with bootstrap values > 70 are marked with circles.

Genome-wide analysis identified 1032 C2H2 genes across 30 basidiomycete species, highlighting the widespread distribution and diversification of this gene family within the phylum. Phylogenetic reconstruction categorized these genes into six distinct clades (Groups I–VI, Figure 1), with substantial variation in gene abundance across groups. Group II was the largest clade, comprising 383 genes (37.1%), followed by Group VI with 259 genes (25.1%), Group IV with 151 genes (14.6%), Group III with 128 genes (12.4%), Group V with 87 genes (8.4%), and Group I with 24 genes (2.3%). This uneven distribution points to functional diversification and lineage-specific expansions during evolution.

**Figure 2:**
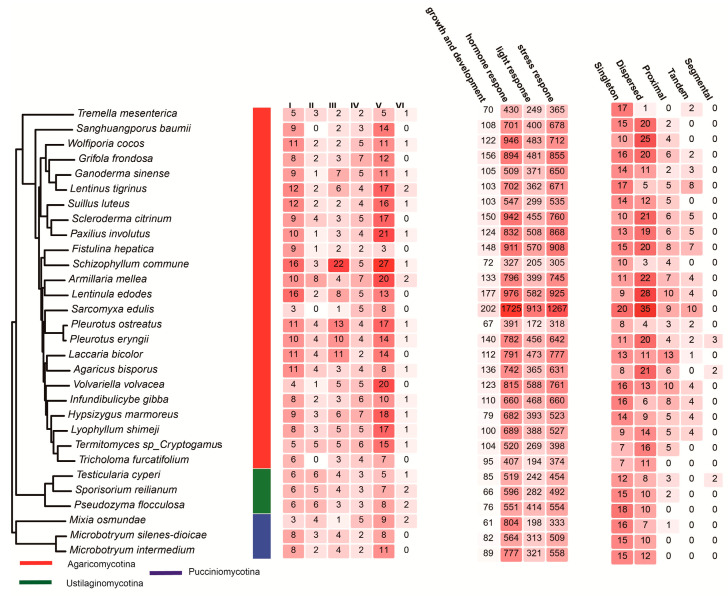
Distribution of C2H2 zinc finger gene types across 30 Basidiomycete species. Left to right, the following can be observed: phylogenetic relationships of species (colored by subphylum); abundance of six C2H2 clades across 30 Basidiomycete species; classification of cis-acting elements in C2H2 gene promoters; duplication modes of C2H2 genes in 30 fungal species. The counts are visualized via a color scale ranging from deep red (highest number) to light red (lowest number).

Interspecific variation in C2H2 gene counts was observed across the 30 species (Figure 2). *Schizophyllum commune* had the highest total (Group I: 16, II: 3, III: 22, IV: 5, V: 27, VI: 1), with significant expansion in Group III. The total gene count in *Armillaria mellea* and *Pleurotus ostreatus* was higher (51 and 50 genes, respectively), while *Fistulina hepatica* and *Sarcomyxa edulis* exhibited minimal representation (17 genes each). *Volvariella volvacea* showed moderate counts (Group I: 4, II: 1, III: 5, IV: 5, V: 20, VI: 0), and *Ganoderma sinense* had relatively few genes (Group I: 9, II: 1, III: 7, IV: 5, V: 11, VI: 1). Notably, the number of genes in Group VI across all species was very small, ranging from 0 to 2.

Linear regression analysis revealed a weak, yet statistically significant, positive correlation between genome size and C2H2 gene count across the 30 Basidiomycota species (y = 22.42 + 0.29x, R^2^ = 0.14, *p* = 0.045; Figure S1).

### 3.2. Physicochemical Properties of C2H2 Zinc Finger Proteins

**Figure 3:**
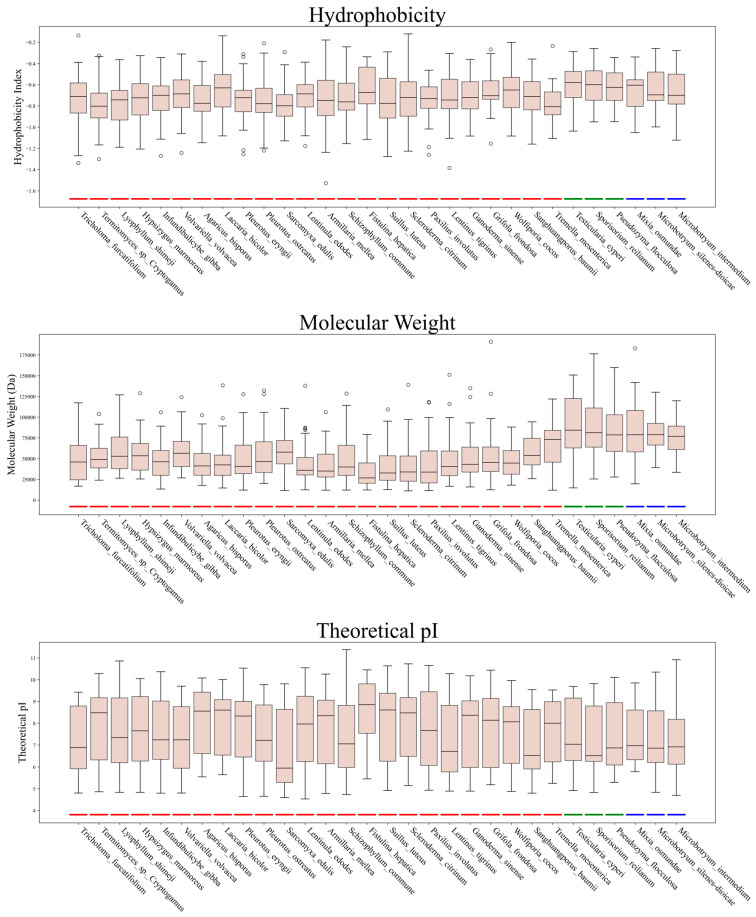
Distribution of C2H2 physicochemical properties in 30 fungal species. Note: Agaricomycotina (red), Ustilaginomycotina (green), and Pucciniomycotina (blue) are highlighted by color.

Comparative analysis of C2H2 zinc finger proteins (ZFPs) across 30 species of Basidiomycota (Agaricomycotina, Pucciniomycotina, and Ustilaginomycotina) revealed substantial divergence in physicochemical properties and subcellular localization (Figure 3, Table S2). Molecular weight (Mw) exhibited significant intra- and inter-subphylum variation, ranging from 31,967.7 kDa in *Fistulina hepatica* (Agaricomycotina) to 88,298.6 kDa in *Testicularia cyperi* (Ustilaginomycotina). Agaricomycotina species displayed moderate Mw (mean ± SD: 49,989.6 ± 26,040.0 kDa), while both Pucciniomycotina (79,016.7 ± 28,261.3 kDa) and Ustilaginomycotina (87,117.1 ± 36,170.7 kDa) exhibited markedly higher values. Theoretical isoelectric points (pI) spanned a broad acidic-to-alkaline range (4.26–10.82), with *Sarcomyxa edulis* (Agaricomycotina) showing the lowest pI (6.69 ± 1.78) and Fistulina hepatica the highest (8.51 ± 1.57). At the subphylum level, Ustilaginomycotina (mean pI = 7.66 ± 1.49) and Pucciniomycotina (7.68 ± 1.36) exhibited slightly lower pI values compared to Agaricomycotina (7.82 ± 1.59). Grand Average of Hydropathicity (GRAVY) scores indicated strong hydrophilicity across all groups (range: −0.121 to −1.529), with *Termitomyces* sp. ‘*cryptogamus*’ (Agaricomycotina) exhibiting the most hydrophilic profile (−0.790 ± 0.208). Notably, *Sporisorium reilianum* and *Testicularia cyperi* (Ustilaginomycotina) showed reduced hydrophilicity (GRAVY: −0.608 ± 0.192 and −0.605 ± 0.196, respectively), suggesting adaptations for membrane-associated functions. Subcellular localization predictions revealed a predominant nuclear localization, with Agaricomycotina exhibiting the highest nuclear localization (672 proteins, 76.7% of total), followed by cytoplasmic distribution (181 proteins, 20.7%). In contrast, Pucciniomycotina and Ustilaginomycotina showed fewer C2H2-ZFPs (76 and 80 total, respectively), though nuclear localization remained predominant (Pucciniomycotina: 62, 81.6%; Ustilaginomycotina: 53, 66.3%). Unique subcellular patterns included a centrosome-localized protein in Pucciniomycotina and two extracellular/mitochondrial proteins in Ustilaginomycotina. High intra-subphylum variability (e.g., Ustilaginomycotina Mw SD = 36,170.7 kDa; *Sarcomyxa edulis* pI SD = 1.78) highlighted functional plasticity within conserved C2H2-ZFP architectures.

### 3.3. Gene Architecture Variations Among Subphyla

**Figure 4:**
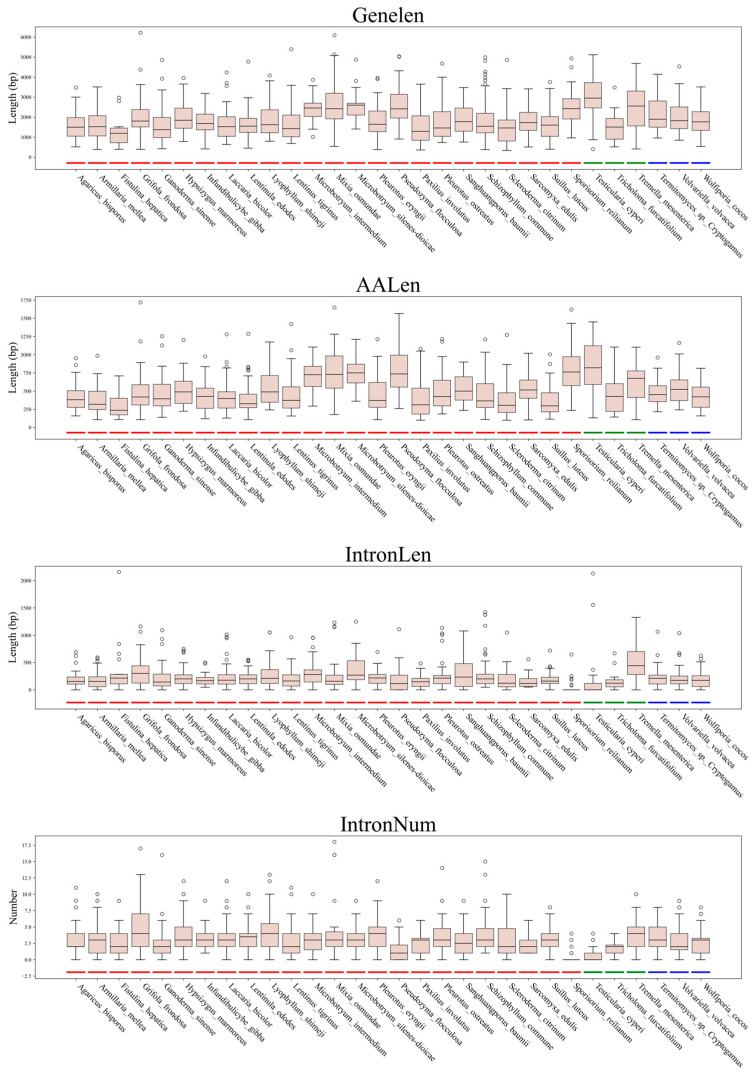
Structural characteristics of C2H2 gene family members in 30 fungal species. Note: Agaricomycotina (red), Ustilaginomycotina (green), and Pucciniomycotina (blue) are highlighted by color.

Analysis of C2H2 gene structural features across the 30 Basidiomycota species revealed distinct subphylum-specific trends (Figure 4, Table S2). Gene length (Genelen) varied significantly, ranging from 1226.1 bp in *Fistulina hepatica* (Agaricomycotina) to 2978.1 bp in *Testicularia cyperi* (Ustilaginomycotina). Amino acid length (AALen) followed a similar pattern, with *Testicularia cyperi* (Ustilaginomycotina) showing the highest mean value (824.6 ± 331.6 residues). Intron characteristics exhibited marked divergence: *Sporisorium reilianum* (Ustilaginomycotina) had the shortest introns (mean length = 54.2 ± 137.8 bp) and the fewest introns per gene (mean number = 0.4 ± 1.0), whereas Pucciniomycotina retained relatively longer introns and more introns per gene. For example, *Mixia osmundae* (Pucciniomycotina), ranked 24th among the species, exhibited moderate intron features (mean length = 297.1 ± 359.9 bp; mean number = 4.5 ± 5.0 per gene). High intra-subphylum variability was evident, such as the Genelen SD of 1335.7 bp in *Mixia osmundae* (Pucciniomycotina) and IntronLen SD of 511.5 bp in *Testicularia cyperi* (Ustilaginomycotina).

### 3.4. Cis-Regulatory Element Distribution in Promoters

A cis-regulatory element is a non-coding DNA region that regulates the transcription of nearby genes by binding to specific proteins. Analysis of cis-regulatory elements in the promoters of C2H2 zinc finger genes across 30 Basidiomycota species revealed distinct subphylum-specific patterns (Figure 2, Table S2).

Growth and Development Elements: Agaricomycotina exhibited the highest average count (118 elements/species), followed by Pucciniomycotina (77 elements/species) and Ustilaginomycotina (76 elements/species). *Schizophyllum commune* exhibited the highest count (202 elements), nearly three times that of the lowest species, *Fistulina hepatica* (67 elements). Notable variation also existed within ecological guilds, with wood decayers like *Pleurotus ostreatus* (148 elements) surpassing symbiotic species such as *Laccaria bicolor* (150 elements).

Hormone Response Elements: Agaricomycotina exhibited a dominant presence (738 elements/species), peaking in *Schizophyllum commune* (1725 elements). Pucciniomycotina averaged 715 elements/species, and Ustilaginomycotina averaged 555 elements/species. *Schizophyllum commune* again led with 1774 elements, far surpassing other species, such as *Sarcomyxa edulis* (327 elements). Pathogenic species like *Sporisorium reilianum* (596 elements) exhibited lower counts compared to saprotrophs such as *Armillaria mellea* (976 elements).

Light Response Elements: Agaricomycotina showed the highest enrichment of light response elements (418 elements/species), particularly in *Schizophyllum commune* (913 elements). Pucciniomycotina averaged 277 elements/species, while Ustilaginomycotina averaged 313 elements/species. *Schizophyllum commune* again dominated (913 elements), contrasting sharply with *Mixia osmundae* (198 elements) and *Testicularia cyperi* (242 elements). Light-responsive capacity was observed to vary independently of trophic modes, with both *Lentinus tigrinus* (saprotroph, 588 elements) and *Pleurotus ostreatus* (pathogen, 570 elements) ranking highly.

Stress Response Elements: Agaricomycotina led with 661 elements/species, while Pucciniomycotina averaged 299 elements/species, and Ustilaginomycotina averaged 500 elements/species. Extreme values ranged from *Schizophyllum commune* (1267 elements) to *Microbotryum intermedium* (55 elements). Notably, stress element abundance did not correlate strictly with pathogenicity as *Sporisorium reilianum* (492 elements) exhibited fewer elements than saprotrophic *Ganoderma sinense* (660 elements).

These four primary classes of cis-regulatory elements were distributed across 33 subclasses, such as light responsiveness, MeJA responsiveness, and salicylic acid responsiveness (Figure S2, Table S3).

### 3.5. Gene Duplication Modes

Analysis of duplication modes revealed subphylum-specific patterns (Figure 2, Table S4). Agaricomycotina lineages, such as *Schizophyllum commune* (dispersed = 35, and tandem = 10) and *Ganoderma sinense* (dispersed = 6, and proximal = 8), exhibited diverse duplication types, with dispersed events predominating (mean = 48% of duplications). In contrast, Pucciniomycotina species like *Microbotryum intermedium* (singleton = 56%) and *Mixia osmundae* (singleton = 67%), as well as Ustilaginomycotina species such as *Sporisorium reilianum* (singleton = 56%), showed a bias towards singleton duplications, reflecting genomic streamlining. Tandem duplications were more prevalent in wood-decaying Agaricomycotina species, including *Volvariella volvacea* (tandem = 23%) and *Pleurotus ostreatus* (tandem = 14%), while segmental duplications were rare (≤7%). Notably, *Armillaria mellea* uniquely exhibited a high proportion of dispersed (55%) and proximal (20%) duplications, indicating adaptive flexibility in its genome.

### 3.6. Synteny and Evolutionary Conservation

**Figure 5:**
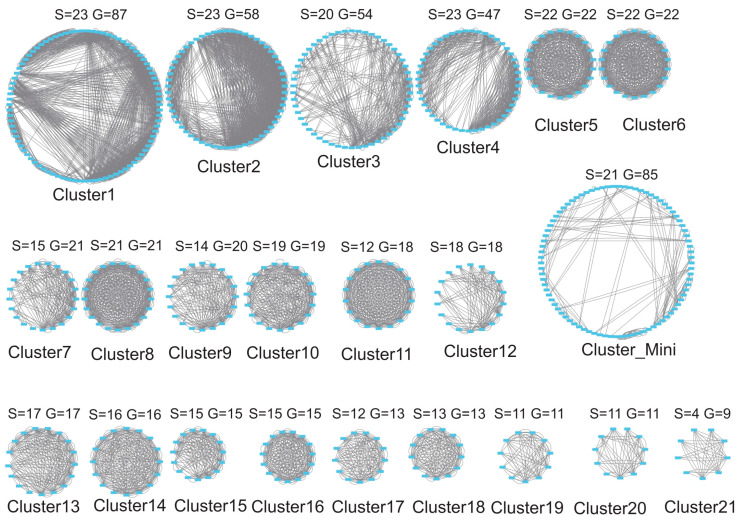
Syntenic relationships of C2H2 genes in 24 Agaricomycotina species. Note: S represents the number of species, and G represents the number of genes.

**Figure 6:**
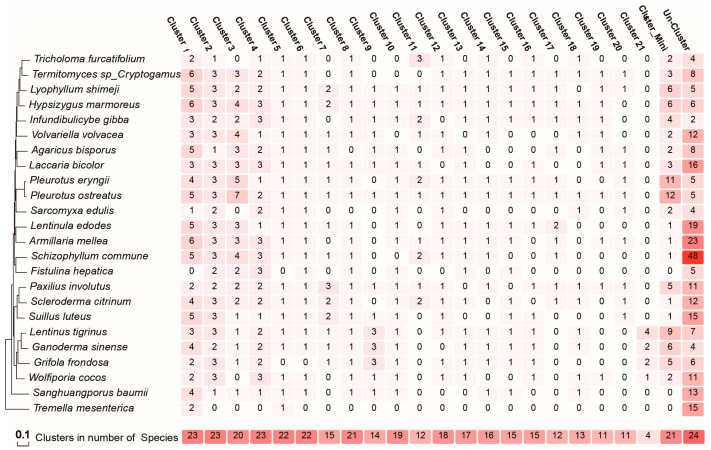
Abundance distribution of C2H2 SynNet communities across 24 fungal species. The counts are visualized via a color scale ranging from deep red (highest number) to light red (lowest number).

Synteny analysis of 24 Agaricomycotina species using JCVI identified 6955 collinear gene pairs (Table S5), revealing extensive conservation patterns of C2H2 zinc finger genes. Visualization of these collinear relationships via Cytoscape delineated 21 syntenic networks (Figure 5), with distinct clusters showing varying degrees of phylogenetic conservation. Notably, Clusters 1–6 were present in ≥90% of the analyzed species (N ≥ 22; Figure 6, Table S6), indicating strong evolutionary constraints on these loci. Synteny network analysis further grouped clusters with low gene counts (Clusters 22–50) into a merged category called Cluster Mini, highlighting their limited representation across species. These conserved clusters likely encode core regulatory functions essential for Agaricomycotina biology.

In contrast, a significant fraction of genes (Un-Cluster) lacked detectable synteny across species, reflecting dynamic evolutionary processes such as lineage-specific gene duplication, neofunctionalization, and genomic rearrangements. The coexistence of conserved and divergent syntenic networks underscores the dual evolutionary trajectory of C2H2 genes in fungi: the preservation of ancestral regulatory modules alongside clade-specific innovations.

Among the 22 primary clusters, 612 conserved C2H2 genes were retained in syntenic regions. Cluster 1 exhibited the highest gene retention (87 genes), followed by progressively smaller clusters, with Cluster 21 containing the fewest conserved genes (9 genes; Table S6). This hierarchical distribution suggests differential evolutionary pressures on C2H2 loci, with larger clusters (e.g., Cluster 1) representing ancestral regulatory hubs critical for conserved cellular processes, while smaller clusters (e.g., Cluster Mini) likely arose from lineage-specific expansions or functional diversification.

### 3.7. Evolutionary Constraints on C2H2 Clusters

**Figure 7:**
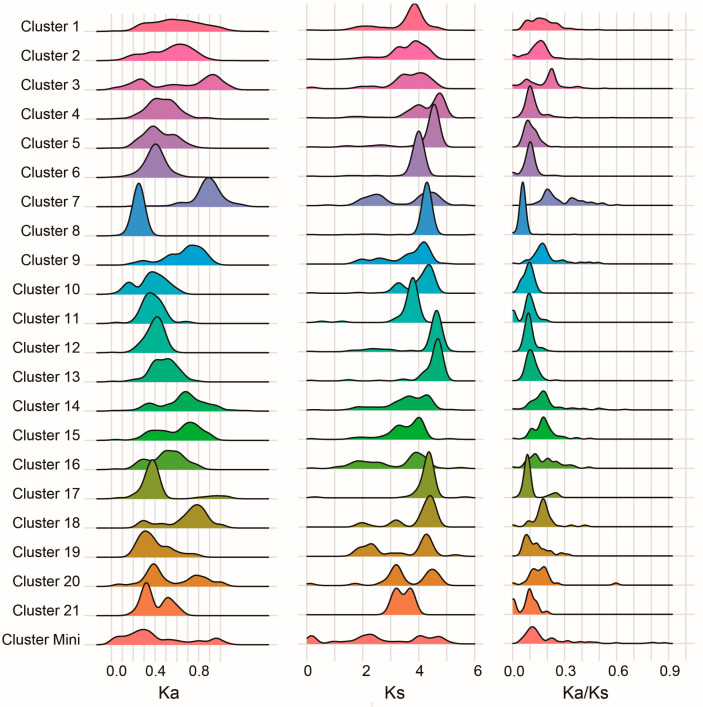
Ka/Ks distribution of syntenic C2H2 gene pairs. Note: Ka/Ks (also written as dN/dS) is the ratio of nonsynonymous substitutions per nonsynonymous site (Ka) to synonymous substitutions per synonymous site (Ks), used to measure selective pressure in protein-coding genes.

To assess evolutionary selection pressures, Ka/Ks ratios were calculated for each synteny network (SynNet) cluster (Figure 7, Table S5). All clusters exhibited Ka/Ks ratios < 1, indicating pervasive purifying selection acting on C2H2 zinc finger genes across Agaricomycotina, consistent with their conserved functional roles. Notably, Cluster 7 exhibited a significantly higher average Ka/Ks ratio than other clusters, suggesting accelerated evolutionary rates, potentially linked to relaxed selective constraints or neofunctionalization.

### 3.8. Expression Patterns of Individual C2H2 Zinc Finger Genes in Transcriptomes of S. edulis

**Figure 8:**
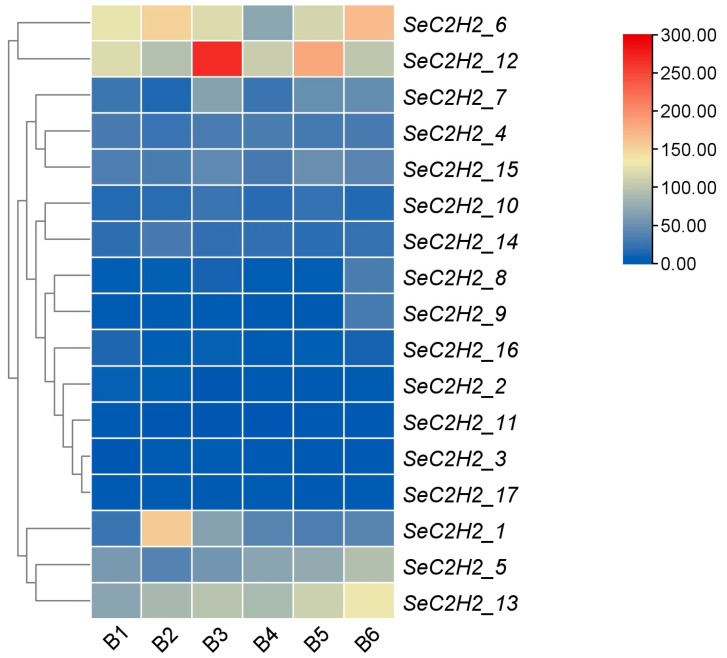
Expression of C2H2 gene family in *Sarcomyxa edulis*.

The transcriptional dynamics of 17 C2H2 zinc finger genes (SeC2H2_1 to SeC2H2_17) in *Sarcomyxa edulis* were analyzed across six developmental stages (B1 to B6) using transcriptomic data (Figure 8). Expression levels (FPKM values) revealed distinct stage-specific patterns among family members. *SeC2H2_1* showed a striking upregulation at B2 (157.897 FPKM), likely linked to low-temperature adaptation, followed by a progressive decline through B6 (40.463 FPKM). *SeC2H2_9* exhibited a 10-fold surge at B6 (31.827 FPKM vs. B1–B5: 2.387–3.87), suggesting its role in fruiting body maturation. *SeC2H2_6* and *SeC2H2_13* maintained high expression throughout development, peaking at B6 (168.143 and 131.48 FPKM, respectively), implicating their involvement in late-stage morphogenesis. In contrast, *SeC2H2_11* remained minimally expressed across all stages (0.433–2.623 FPKM), indicating potential functional redundancy or suppression. Notably, *SeC2H2_12* and *SeC2H2_6* displayed transient peaks at B3 (268.26 and 120.15 FPKM, respectively), coinciding with primordia initiation, while *SeC2H2_8* sharply increased at B6 (33.737 FPKM), possibly regulating post-harvest processes. These findings demonstrate that the C2H2 family in *S. edulis* orchestrates developmental transitions through temporally partitioned expression programs.

## 4. Discussion

C2H2-type zinc finger genes were most abundant in Agaricomycotina, whereas species in Pucciniomycotina and Ustilaginomycotina exhibited significantly lower gene counts. This pattern aligns with findings from studies on cytochrome P450 monooxygenases (P450s): Armillaria mellea in Agaricomycotina harbored the largest P450 repertoire (267 genes), greatly outnumbering those in Pucciniomycotina species (including *Melampsora laricis-populina*, *M. lini*, *Mixia osmundae*, and *Puccinia graminis*) and Ustilaginomycotina species (such as *Ustilago maydis*, *Sporisorium reilianum*, and *Tilletiaria anomala*) [21]. This parallel reduction in both gene families suggests potential evolutionary constraints or lineage-specific genomic streamlining in *M. osmundae* [4,22].

Subphylum-specific physicochemical properties further illuminate adaptive strategies. The alkaline pI bias in pathogenic Ustilaginomycotina/Pucciniomycotina (pI 8.3–8.6) likely optimizes nuclear localization of transcription factors during host infection, paralleling trends observed in pathogenic Ascomycota effector proteins [23,24]. Conversely, the acidic pI range in Agaricomycotina saprotrophs (pI 4.3–5.8) may enhance protein solubility for extracellular enzymatic activities, similar to lignocellulolytic CAZymes in white-rot fungi. Striking structural contrasts, such as *Armillaria mellea*’s extreme hydrophobicity (GRAVY = 0.19) and molecular weight (49 kDa), suggest neofunctionalization events that enable niche-specific adaptations, potentially through protein–protein interactions in lignocellulose degradation [25].

Gene duplication, including whole-genome duplication (WGD), tandem duplication, segmental duplication, and transposition, is a major driver of genome evolution and the emergence of novel genes and species [26]. Both WGD and tandem duplication have been shown to significantly influence genome evolution and species diversification. In this study, Agaricomycotina lineages, such as *Schizophyllum commune* and *Ganoderma lucidum*, exhibited a predominance of dispersed duplication (mean = 48%). Previous studies have linked tandem duplication to the expansion of genes involved in biotic and abiotic stress responses [27,28,29]. Freeling et al. [26] further demonstrated that tandem duplication often amplifies genes at the termini of metabolic pathways or those insensitive to dosage effects. Our findings suggest that the C2H2 zinc finger gene family in Basidiomycota retains a higher proportion of segmentally duplicated genes and a lower proportion of tandemly duplicated genes. This indicates that C2H2 zinc finger genes are crucial for the life processes of Agaricomycotina, and their loss may disrupt metabolic stability in Basidiomycota. Additionally, only a small proportion of C2H2 zinc finger genes are involved in stress responses, highlighting their functional specificity beyond environmental adaptation.

Homologous gene pairs exhibit divergent evolutionary trajectories in terms of gene retention and functional divergence, leading to distinct fates among duplicated copies [30]. Subsequent genetic differentiation can result in nonfunctionalization, subfunctionalization, neofunctionalization, or conservation. Molecular evolutionary rate analysis of paralogs and orthologs revealed that certain genes consistently undergo positive selection, suggesting accelerated evolutionary rates and potential functional diversification. In this study, all C2H2 homologous gene pairs displayed Ka/Ks ratios < 1. Specifically, Clusters 1–12 showed strong purifying selection (Ka/Ks = 0.15–0.22), while Clusters 19–20 exhibited nearly neutral evolution (Ka/Ks = 0.85–0.89). These findings imply that fungal C2H2 genes are under intense purifying selection (negative selection), existing in an evolutionary “steady state” with highly optimized functions and limited potential for further adaptive mutations [31]. Given their likely involvement in core biological processes, these genes should be prioritized for functional characterization.

To further investigate the variation and conservation of C2H2 genes, a statistical analysis of intron numbers across species was performed. Overall, the distribution of intron numbers within each species was largely consistent, with some exceptions showing significant divergence. For example, in Agaricus bisporus, 87.1% of the 31 C2H2 genes predominantly contained 0–5 introns. Notably, C2H2 genes in *Sporisorium reilianum* and *Testicularia cyperi* (subphylum Ustilaginomycotina) exhibited particularly low intron counts (ranging from 0 to 4), with the highest proportions (77.8% and 64%, respectively) lacking introns entirely. This pattern suggests strong evolutionary conservation, consistent with previous findings [1]. Collectively, these results emphasize the structural conservation of the C2H2 gene family within species while revealing limited structural variation in a subset of genes. Such variation may result from exon–intron reorganization during evolution, potentially driven by functional selection for critical gene segments [32].

The distribution and diversity of cis-regulatory elements in promoter sequences are crucial for modulating gene expression and functional specialization [33]. Our analysis revealed that C2H2-ZFP genes are enriched with cis-elements linked to growth and development, light responsiveness, and stress responses. Notably, subclasses associated with light responsiveness, MeJA (methyl jasmonate) responsiveness, and salicylic acid responsiveness constituted 47.6% of all identified cis-regulatory elements, underscoring the potential significance of C2H2-ZFPs in hormone signaling and light adaptation processes in Basidiomycota. These findings align with the well-documented roles of C2H2-ZFPs in stress and environmental adaptation across various species [34].

The stage-specific expression patterns of the C2H2 zinc finger gene family in *Sarcomyxa edulis* uncovered in this study provide critical insights into their potential roles in coordinating developmental transitions and environmental adaptation. The dramatic upregulation of *SeC2H2_1* during B2 (low-temperature-stimulated mycelial colonization) aligns with previous reports that C2H2 transcription factors mediate cold stress responses in fungi by regulating membrane fluidity and antioxidant pathways [35]. Its subsequent decline in later stages suggests a transient role in acclimatizing to abiotic stress rather than sustaining growth. Similarly, the abrupt activation of *SeC2H2_9* and *SeC2H2_8* at B6 (mature fruiting body) correlates with the demand for cell wall remodeling and secondary metabolite synthesis during sporulation, mirroring functions observed in Aspergillus C2H2 regulators [36,37]. The sustained high expression of *SeC2H2_6* and *SeC2H2_13* across all stages implies their involvement in core developmental processes, such as hyphal tip growth and nutrient acquisition [38]. This is consistent with their homologs in *Pleurotus ostreatus*, which are essential for maintaining mycelial vitality under fluctuating resource availability [39]. Conversely, the persistently low expression of *SeC2H2_11* may reflect functional redundancy or epigenetic silencing, a phenomenon documented in plant C2H2 families under stable environmental conditions [40]. Notably, the transient peaks of *SeC2H2_12* and *SeC2H2_7* at B3 (primordia initiation) coincide with the activation of morphogenetic signaling pathways. We hypothesize that these genes act as molecular switches to trigger primordia differentiation, potentially through interactions with light-responsive or cAMP-dependent regulators [41,42]. Future studies integrating CRISPR-Cas9 mutagenesis and transcriptome profiling of knockout strains are required to validate these functional assignments and elucidate downstream targets.

## 5. Conclusions

Our phylogenomic analysis of 1032 C2H2 genes across Basidiomycota delineated their evolutionary trajectory into six clades with distinct functional roles. The conserved regulatory core (Group II) and clade-specific expansions in wood decayers (Group III) and edible fungi (Group V) highlight adaptive specialization driven by ecological niches. Notable contrasts in genomic architecture—saprotrophs maintaining intron-rich, structurally complex genes versus pathogens exhibiting streamlined genomes—reflect divergent survival strategies. Synteny network analysis further reveals evolutionary forces shaping this gene family: ancestral clusters, under strong purifying selection (Ka/Ks < 0.25), preserve essential regulatory functions, while peripheral clusters, approaching neutral evolution (Ka/Ks = 0.73), may act as innovation hubs. These dual evolutionary modes—the conservation of core networks and plasticity in adaptive modules—position C2H2 zinc fingers as molecular fulcrums balancing genomic stability with ecological adaptability. The expression profiles revealed significant stage-specific variations among family members in *Sarcomyxa edulis*. Our findings enhance the mechanistic understanding of fungal genome evolution and offer a framework for utilizing these regulators in crop disease management and lignocellulosic bioconversion engineering.

## Data Availability

The original contributions presented in the study are included in the article/Supplementary Material, further inquiries can be directed to the corresponding authors. The original data presented in the study are openly available in [NCBI] at [https://www.ncbi.nlm.nih.gov/bioproject/PRJNA739377/, accessed on 25 February 2025], [PRJNA739377].

## Supplementary Materials

The following supporting information can be downloaded at https://www.mdpi.com/article/10.3390/jof11070487/s1. Figure S1. Positive correlation between genome size and C2H2 zinc finger gene abundance in Basidiomycota; Figure S2. Detailed distribution of cis-acting elements in C2H2 gene promoters; Table S1. Taxonomic list of 30 fungal species analyzed in this study; Table S2. Comprehensive physicochemical properties of C2H2 gene family members. Note: This includes gene ID, protein ID, taxonomic classification, chromosomal location (start/end sites), amino acid length, molecular weight, theoretical isoelectric point (pI), hydropathicity, and subcellular localization; Table S3. Promoter regions; Table S4. Replication types; Table S5. Collinearity analysis; Table S6. Collinearity network-conserved genes.
