# Supplementary figures and images for: Evolutionary Dynamics and Functional Bifurcation of the C2H2 Gene Family in Basidiomycota
Source: J Fungi (Basel). 2025 Jun 27;11(7):487. doi: 10.3390/jof11070487 (PMC12295980; doi:10.3390/jof11070487)

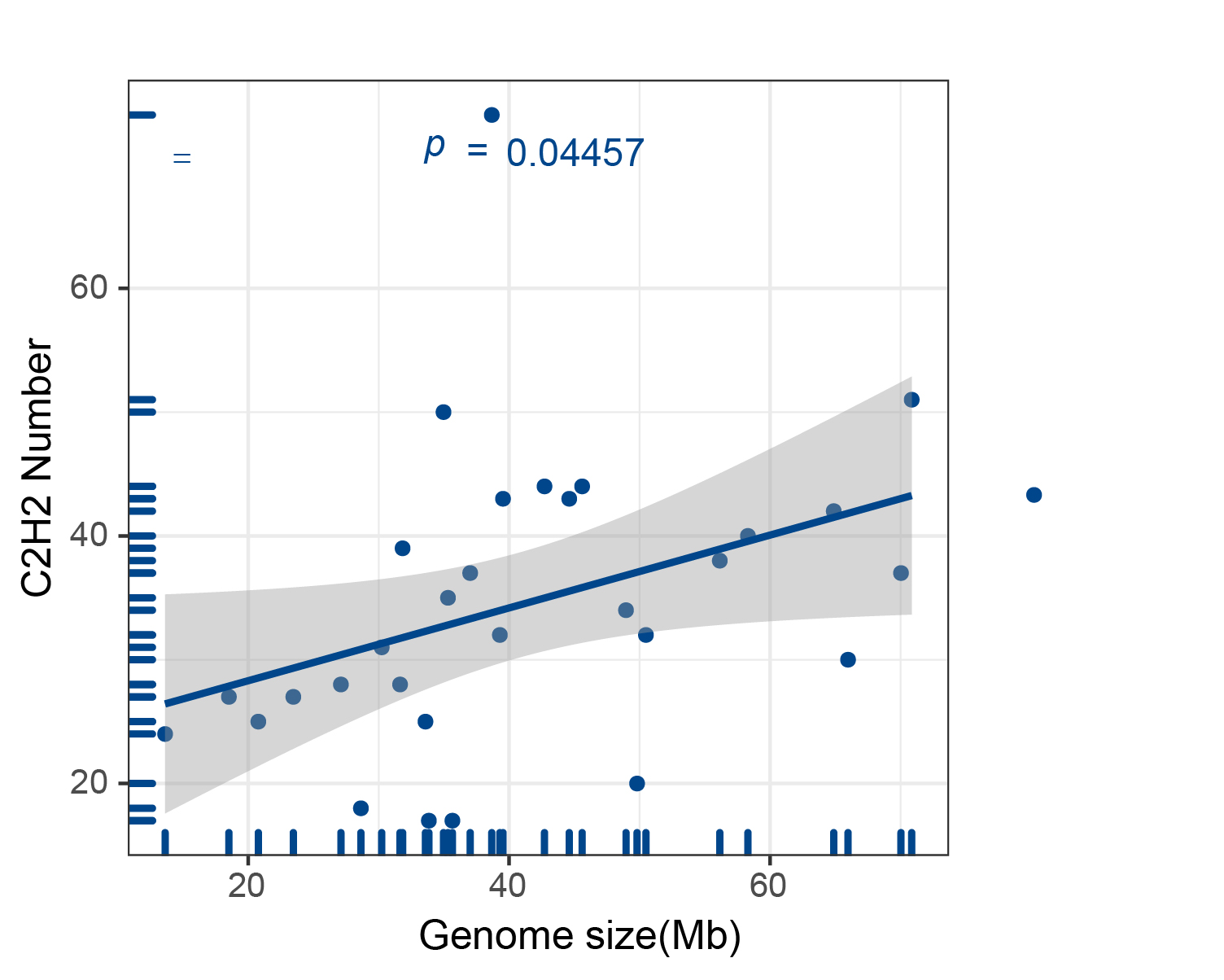

Supplement: Supplementary file 1 [file jof-11-00487-s001.zip › Figure S1.jpg]

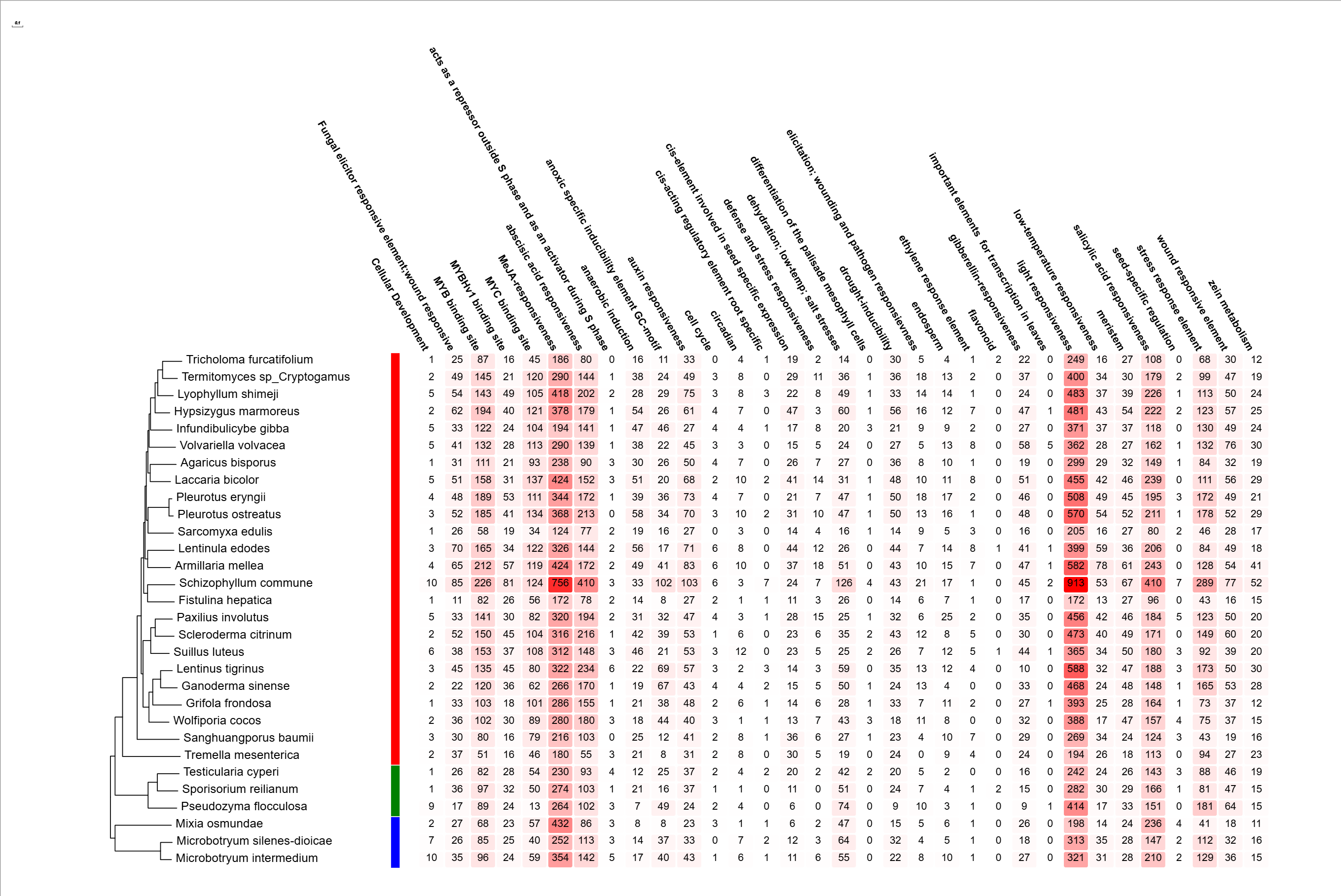

Supplement: Supplementary file 1 [file jof-11-00487-s001.zip › Figure S2.jpg]
